# Elucidating the Role of Human ALAS2 C-terminal Mutations Resulting in Loss of Function and Disease

**DOI:** 10.1021/acs.biochem.4c00066

**Published:** 2024-06-18

**Authors:** Jessica L. Taylor, Pedro H. Ayres-Galhardo, Breann L. Brown

**Affiliations:** ^†^Department of Biochemistry, ^‡^Center for Structural Biology, Vanderbilt University School of Medicine, Nashville, Tennessee 37232, United States

## Abstract

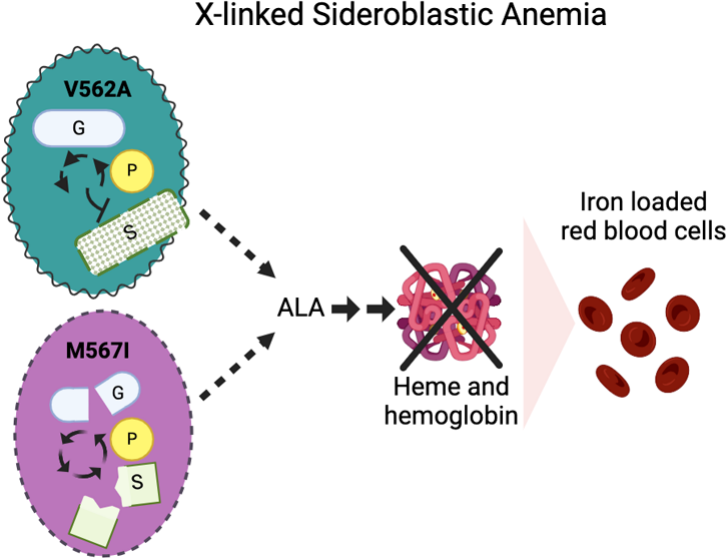

The conserved enzyme aminolevulinic acid synthase (ALAS) initiates heme biosynthesis in certain bacteria and eukaryotes by catalyzing the condensation of glycine and succinyl-CoA to yield aminolevulinic acid. In humans, the ALAS isoform responsible for heme production during red blood cell development is the erythroid-specific ALAS2 isoform. Owing to its essential role in erythropoiesis, changes in human ALAS2 (hALAS2) function can lead to two different blood disorders. X-linked sideroblastic anemia results from loss of ALAS2 function, while X-linked protoporphyria results from gain of ALAS2 function. Interestingly, mutations in the ALAS2 C-terminal extension can be implicated in both diseases. Here, we investigate the molecular basis for enzyme dysfunction mediated by two previously reported C-terminal loss-of-function variants, hALAS2 V562A and M567I. We show that the mutations do not result in gross structural perturbations, but the enzyme stability for V562A is decreased. Additionally, we show that enzyme stability moderately increases with the addition of the pyridoxal 5′-phosphate (PLP) cofactor for both variants. The variants display differential binding to PLP and the individual substrates compared to wild-type hALAS2. Although hALAS2 V562A is a more active enzyme *in vitro*, it is less efficient concerning succinyl-CoA binding. In contrast, the M567I mutation significantly alters the cooperativity of substrate binding. In combination with previously reported cell-based studies, our work reveals the molecular basis by which hALAS2 C-terminal mutations negatively affect ALA production necessary for proper heme biosynthesis.

## Introduction

Heme mediates critical biological processes, such as drug metabolism in the liver, oxygen transport in red blood cells, and electron transport in mitochondria.^1^ Erythropoiesis relies on controlled and effective heme production to accommodate the high demand for developing red blood cells. As a result, aberrations in any step of heme biosynthesis during late erythropoiesis can result in several blood diseases.^2−5^ Aminolevulinic acid synthase (ALAS) catalyzes the first and rate-limiting step of heme biosynthesis, in which glycine and succinyl-CoA condense into aminolevulinic acid (ALA).^6−9^ ALAS is conserved among α-proteobacteria and nonplant eukaryotes, where it is transported to the mitochondrial matrix to initiate heme biosynthesis (reviewed in ref (10)). Vertebrates evolved to have two ALAS isoforms encoded by separate genes.^11,12^ ALAS1 is a ubiquitously expressed isoform, and ALAS2 is specific to erythroid progenitor cells.^12,13^ Coinciding with the constant requirement for heme during erythropoiesis, ALAS2 initiates the production of approximately 85% of all heme for hemoglobin in humans.^14^

Currently, there are more than 90 reported *ALAS2* mutations that underlie two separate diseases.^15,16^ X-linked protoporphyria (XLP) arises from deletions or frameshift truncations specifically affecting the hALAS2 C-terminal extension, which cause a gain of function.^3,17^ Conversely, X-linked sideroblastic anemia (XLSA) occurs from missense mutations that result in ALAS2 loss of function.^15,18^ A consequence of ALAS2 loss of function is the toxic accumulation of iron in the mitochondria of erythroid progenitors due to diminished production of porphyrin precursors accompanied by sustained mitochondrial iron transport.^18,19^ Iron delivery to erythroid precursors occurs primarily in developing normoblasts, with the maximal expression of transferrin receptors 1 and 2 occurring in intermediate normoblasts.^1,20^ Thus, a majority of iron import occurs upstream of ALAS2 activation. Also, ineffective erythropoiesis is an additional signal to increase iron accumulation due to the suppression of hepcidin, the master regulator of iron homeostasis.^21−25^ Consequently, ALAS2 dysfunction and a resulting decrease in protoporphyrin IX production leads to the development of iron-overloaded erythroblasts.^26−28^ In severe cases, the accumulation of iron can cause irreversible organ damage and fatality.^29,30^ Significantly, disease severity is difficult to predict based solely on the presence of a specific genetic mutation due to differences in metabolism, age, diet, and sex.^31−33^

ALAS enzymes from eukaryotes contain a C-terminal extension that is absent in the bacterial homologues.^34,35^ The hALAS2 C-terminal extension is comprised of the final 42 amino acids and is often referred to as the ALAS2 autoinhibitory domain because deletion of this region results in enzyme hyperactivity that underlies XLP.^17,36^ Despite this phenotype, several loss-of-function point mutations occur in the hALAS2 C-terminal extension. Additionally, multiple studies suggest that hALAS2 is part of a larger macromolecular complex, or metabolon, that would serve to modulate heme production.^37,38^ ALAS2 interactors include the TCA cycle proteins succinyl-CoA synthetase (SCS), α-ketoglutarate dehydrogenase, and the ATP-dependent unfoldase ClpX.^39−42^ SCS was reported to interact specifically with the ALAS2 C-terminus and certain XLSA variants correlate with a loss of this complex.^40^ However, whether changes in protein–protein interactions are drivers of disease is currently unknown. Thus, there remains much to be uncovered regarding how perturbations in this key enzyme regulatory region shift hALAS2 activity in opposing ways to alter heme production.

Two previously reported hALAS2 XLSA variants that impact the C-terminal extension are Val562Ala (V562A, c.1685T > C) and Met567Ile (M567I, c.1701G > A).^43^ These mutations were identified in independent male probands that exhibited iron overload and reduced hemoglobin levels. Although both mutations affect hydrophobic residues in the C-terminus, each displayed unique characteristics *in vitro* and *in situ*. The V562A variant had higher *in vitro* activity but a significantly shorter cellular half-life compared to wild-type hALAS2 (WT). However, the M567I variant displayed lower activity but higher cellular stability.^43^ Having been established as bona fide disease alleles, we sought to determine the molecular basis for altered enzyme function. We discovered that although hALAS2 V562A has favorable turnover rates, its catalytic efficiency is diminished upon succinyl-CoA binding. Additionally, we observed that V562A has a lower thermal stability than WT or M567I, which is consistent with the previously reported cellular data that suggests this mutation is destabilizing. In contrast, we found that hALAS2 M567I binds both substrates with negative cooperativity, which may contribute significantly to the decreased activity underlying the disease. Importantly, this work highlights how diverse and combinatorial mechanisms leading to hALAS2 dysfunction can result in pathology.

## Materials and Methods

### Protein Expression and Purification

DNA-encoding hALAS2_54–587_ (UniProt ID P22557) was cloned into the pET28b vector with a ULP1 protease cleavable N-terminal His_6_-SUMO tag and expressed in BL21-Codon Plus (DE3)-RIL cells (Agilent Technologies) in LB media with 50 μg/mL kanamycin. Site-directed mutagenesis was performed on hALAS2_54–587_ using complementary primers (Table S1) and confirmed by sequencing. All variants were transformed into BL21(DE3) RIL competent cells and single-colony 50 mL LB inoculations were grown overnight at 37 °C. The cultures were expanded by placing 10 mL of the overnight cultures into 1 L LB with 50 μg/mL kanamycin. The cultures were grown at 37 °C and induced at an OD_600_ of 0.6–0.8 with 0.5 mM IPTG for 4 h at 22 °C. Cultures were then centrifuged and cell pellets were stored at −80 °C.

Cells were lysed with a high-pressure homogenizer and centrifuged at 30,000*g* for 20 min at 4 °C. The clarified cell extract was incubated at 4 °C with Ni^2+^-NTA agarose resin (Qiagen) pre-equilibrated with lysis buffer (25 mM HEPES, pH 8.0, 400 mM NaCl, 100 mM KCl, 20 mM imidazole, 10% glycerol, and 1 mM DTT). The protein was eluted with elution buffer (25 mM HEPES, pH 8.0, 400 mM NaCl, 100 mM KCl, 250 mM imidazole, 10% glycerol, 1 mM DTT). The His_6_-SUMO tag was removed by overnight incubation at 4 °C with ULP1 protease while dialyzing against 25 mM HEPES, pH 7.0, 150 mM KCl, 10% glycerol, 1 mM DTT. Following dialysis, the cleaved His_6_-SUMO tag was removed with a second Ni^2+^-NTA purification. The eluant fractions were concentrated and applied to a Superdex 200 pg 16/600 column at 4 °C pre-equilibrated in Gel Filtration Buffer (25 mM HEPES, pH 7.0, 150 mM KCl, 10% glycerol, 0.5 mM TCEP). Eluted protein fractions were pooled and concentrated to 10–20 mg/mL.

The hALAS2_54–587_ apoenzymes were prepared by overnight incubation of the protein after the second Ni^2+^-NTA purification step in stripping buffer (0.1 M potassium phosphate, pH 7.5, 10% glycerol, 1 mM DTT) with 5 mM hydroxylamine HCl. Following gel filtration, the protein fractions were pooled and concentrated to 10–20 mg/mL.

### Enzyme Activity Assays

Enzyme activity was measured with a discontinuous colorimetric activity assay as previously described with adaptations.^44^ The reaction was initiated by combining 50 mM potassium phosphate (pH 7.0), 1 mM DTT, 10 mM MgCl_2_, varying concentrations of glycine and succinyl-CoA, 10 μM PLP, and 100 nM (6 μg/mL) purified enzyme (175 μL total). The reaction was incubated at 37 °C for 15 min (previously optimized for linear ALA formation with enzyme concentration) and terminated by adding 100 μL of prechilled 10% trichloroacetic acid.^45^ The mixtures were centrifuged at 13,000*g* for 5 min to remove protein, and 240 μL of the resulting supernatant was added to 240 μL of 1 M sodium acetate, pH 4.6, followed by the addition of 20 μL of acetylacetone. The samples were boiled at 100 °C for 10 min and then cooled for 15 min. Three 100 μL aliquots per reaction (technical replicates) were dispensed into a 96-well clear-bottom plate and further derivatized with the addition of 100 μL of modified Ehrlich’s reagent. The absorbance was monitored at 553 nm for 25 min with a microplate reader (Thermo Fisher Scientific). Absorbance values collected at the spectral maxima for each sample were converted to molar quantities of ALA using an extinction coefficient of 60,400 M^–1^cm^–1^. To determine kinetic parameters (*V*_max_, *K*_m_, and *k*_cat_), 5–100 mM glycine was combined with either 300 μM (WT and M567I) or 1.25 mM succinyl-CoA (V562A) and 50–1000 μM succinyl-CoA was combined with 100 mM glycine. The data were fit with either a Michaelis–Menten or Allosteric Sigmoidal model and statistical significance was determined using a one-way ANOVA (GraphPad Prism). All experiments were performed with a minimum of three biological replicates, each with three technical replicates.

### UV–Visible Absorbance and Fluorescence Spectroscopy

Absorbance measurements for hALAS2_54–587_ variants were obtained using a Molecular Devices SpectraMax M2 microplate reader with an ultramicro quartz cuvette at room temperature. Absorbance scans were executed from 200 to 500 nm with 40 μM protein in Gel Filtration Buffer. Experimental scans were normalized by subtracting buffer-only spectra. The population of PLP tautomer bound was determined by calculating the area under the curve (AUC) from 313–355 nm for the substituted aldimine and 380–480 nm for the ketoenamine species. Data represent the mean of 4–6 biological replicates and statistical significance was determined using a one-way ANOVA.

The fluorescence emission spectra of WT hALAS2_54–587_ and hALAS2_54–587_ variants were monitored in a black 384-well plate with a microplate reader. Each protein was diluted to 30 μM with 1× PBS. PLP tautomers were visualized via excitation at 326 and 424 nm for the substituted aldimine and ketoenamine, respectively. Experimental scans were normalized by subtracting buffer-only spectra. Data represent the mean of three biological replicates and statistical significance was determined using a one-way ANOVA.

### PLP Binding Affinity Assays

For kinetic analyses, the apoenzyme preparations were titrated with increasing concentrations of PLP and measured for activity as described above. The data were fit with a Michaelis–Menten model and statistical significance was determined using a one-way ANOVA. All experiments were performed with a minimum of three biological replicates, each with three technical replicates.

### Protein Unfolding Assays

The melting temperature of hALAS2_54–587_ variants was assayed with and without excess PLP using nano differential scanning fluorimetry (Prometheus NT.Plex). Proteins were diluted to 16.9 μM in 25 mM Hepes, pH 7.0, 100 mM KCl, 10% glycerol, and 0.5 mM TCEP and aspirated into Prometheus NT.48 standard capillaries. Fluorescence intensities at 330 and 350 nm were measured from 25 to 90 °C with a ramp rate of 1 °C/min. For experiments containing PLP, proteins were incubated with 169 μM PLP on ice for 10 min prior to data acquisition. The unfolding temperature (*T*_m_) was determined based on the inflection point of the 350/330 nm absorbance ratio as a function of increasing temperature. Data represent the mean of three biological replicates and statistical significance was determined using a one-way ANOVA.

### Circular Dichroism Polarimetry

Far-ultraviolet (UV) CD spectra were measured using a JASCO J-810 spectrometer. The proteins were diluted to 8.0 μM (WT and M567I) or 6.0 μM (V562A) in 10 mM potassium phosphate pH 7.0, 100 mM KCl, and 0.5 mM TCEP and placed in a quartz cuvette with 1.0 mm path length. Spectra were recorded in continuous scanning mode at 25 °C, from 250 to 190 nm at a scan speed of 20 nm/min with 0.5 nm data pitch and 1.0 nm bandwidth. Three spectra were accumulated and averaged for each sample. Experimental scans were normalized by subtracting buffer-only spectra. Data are reported as mean molar ellipticity.

### Protein Modeling

Computational models of hALAS2 variants were generated using the AlphaFold tool in ChimeraX and the ColabFold platform.^46^ Two copies of either the WT or mutant full-length sequence (residues 1–587) were used as the template query. Five models were generated for each prediction, and the highest-scoring model was chosen for further evaluation. All model figures were generated using PyMOL.^47^

## Results and Discussion

### *In Silico* Analysis of hALAS2 C-terminal XLSA Missense Variants

**Figure 1:**
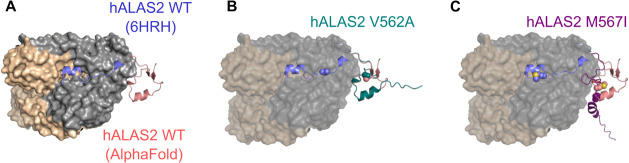
Experimental and computational hALAS2 models display C-terminal flexibility. (A–C) The catalytic core (residues 140-547) of human ALAS2 is shown as a surface representation with one protomer in tan and the symmetry-related protomer in gray. The C-terminal extension (residues 548-587) of each model is shown in cartoon representation and colored as indicated. The flexible N-terminal extensions (residues 1-139) for all AlphaFold models are not shown for clarity. (A) The overlay of the WT human ALAS2 homodimer from the crystal structure (PDB ID 6HRH) and AlphaFold predicted model. The C-terminal extensions are colored blue and pink, respectively. (B) Superposition of the hALAS2 crystal structure (blue) and the AlphaFold models of hALAS2 WT (pink) and hALAS2 V562A (teal). Residue 562 is shown as spheres. (C) Superposition of the hALAS2 crystal structure (blue) and the AlphaFold models of hALAS2 WT (pink) and hALAS2 M567I (purple). Residue 567 is shown as spheres. Complete AlphaFold models colored based on pLDDT score are shown in Figure S1.

The hALAS2 gain-of-function domain is mapped to residues ∼540-580 of the C-terminal extension.^36^ Although this domain is specifically perturbed in all known XLP variants, there are currently 10 reported loss-of-function missense mutations located in this domain, including hALAS2 V562A and M567I. With the increasing predictive power of computational and bioinformatics tools, we revisited the possibility of structural changes due to each mutation that may reveal clues regarding enzyme dysfunction. We compared the best models produced with either hALAS2 WT, V562A, or M567I mutations using AlphaFold Multimer.^46,48,49^ The crystal structure of hALAS2 (PDB 6HRH) was also included for comparison.^45^ The hALAS2 WT C-terminal extension adopts a drastically different orientation in the AlphaFold model compared to the crystal structure (Figure 1A). The AlphaFold prediction of this region displayed low confidence (average pLDDT ∼ 60) and the last nine C-terminal residues of the crystal structure were disordered, indicating significant flexibility in the position of this domain (Figures 1A, S1). The AlphaFold models of the hALAS2 variants displayed similarly low confidence in this domain (pLDDT ∼ 52 and 47 for V562A and M567I, respectively); however, the position of each domain was unique compared to the other suggesting mild perturbations within the region among the variants (Figure 1B,C). In addition to the structure prediction, AlphaMissense was used to predict variant pathogenicity.^50^ Out of the 10 C-terminal XLSA variants, only two were predicted to be pathogenic: hALAS2 L545Q and M567I, with M567I having the highest confidence score. The predicted pathogenicity of the other variants was either ambiguous or benign, including V562A. Thus, computational analysis of the hALAS2 C-terminus alone did not confidently yield insight into a potential molecular basis for enzyme dysfunction as it is probable that the extended C-terminus adopts an ensemble of biologically relevant conformations.^51^

### Hydrophobic C-terminal Mutations Differentially Impact *In Vitro* Protein Stability

**Figure 2:**
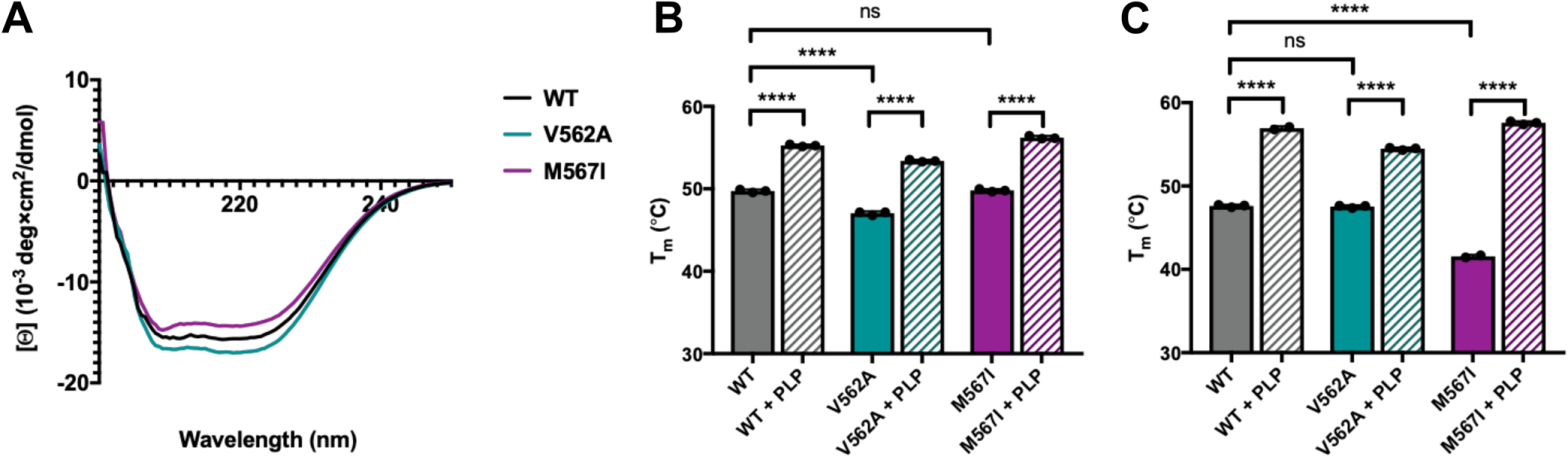
Impact of C-terminal mutations on enzyme structure and stability. (A) Circular dichroism polarimetry analysis of WT, V562A, and M567I hALAS2 variants. (B, C) The unfolding temperatures of holo hALAS2 variants (B) or apo hALAS2 variants (C) assayed by differential scanning fluorimetry in the absence (solid bars) or presence (hatched bars) of exogenous PLP (****p < 0.0001, ns: not significant).

A previous report identified that both V562A and M567I had significantly different stability in HEK293 cells, in which hALAS2 V562A had a shorter half-life than WT, and M567I was significantly stabilized compared to WT.^43^ To determine the *in vitro* properties that may account for the differential stability, we expressed and purified mature hALAS2 (residues 54–587) and both C-terminal variants in both the active, cofactor-bound form (holo) and inactive form (apo) from *E. coli*. All proteins were well-folded as purified and the variants maintained overall secondary structure in comparison to WT hALAS2 (Figure 2A). Additionally, the variants were purified with the C-terminal extension intact as assayed by Western blot (Figure S2) and mass spectrometry. To determine if the mutations impacted *in vitro* protein stability, we measured the protein unfolding temperature (*T*_m_) using differential scanning fluorimetry. The WT and M567I holoenzymes had similar *T*_m_ values, whereas V562A had a *T*_m_ approximately 3 °C lower than the other constructs, indicating destabilization of this variant (Figure 2B). The addition of excess PLP resulted in a significant increase in protein stability of ∼6 °C for all variants (Figure 2B). The WT and M567I apoenzymes had decreased *T*_m_ values compared to their holo counterparts. Significantly, the apo M567I variant displayed the largest decrease in *T*_m_ compared to the holoenzyme and the largest response to the addition of PLP, which led to a 16 °C increase in *T*_m_ (Figure 2C). Exogenous PLP stabilized the WT apoenzyme by approximately 9 °C, and the V562A variant showed the lowest PLP-induced stability, resulting in a 7 °C increase in *T*_m_. Interestingly, the M567I mutation was predicted to be pathogenic, whereas the V562A variant was predicted to be benign. Thus, the experimental data aligned with the predicted outcome only in reference to the apoenzyme stability. However, the *in vitro* thermal stability analysis was consistent with the observed cellular data, which showed that V562A was a destabilizing mutation. Since pyridoxine (which is metabolized to PLP) supplementation is a common treatment for XLSA, these data support the role of PLP-mediating enzyme stability.

### ALAS2 C-terminus Impacts the Mode of PLP Cofactor Binding

**Figure 3:**
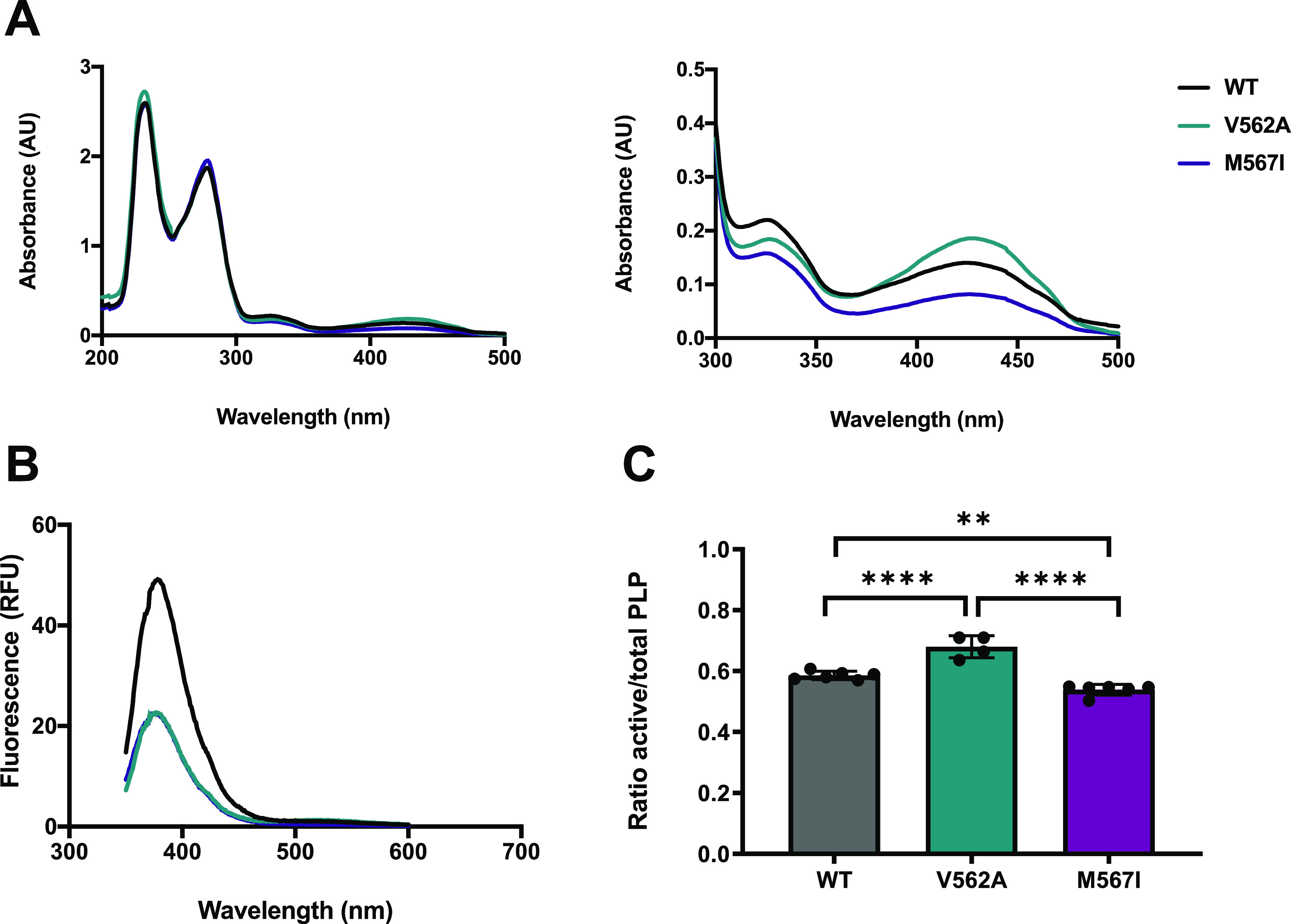
Absorbance and fluorescence spectroscopy reveal the mode of steady-state PLP binding. (A) UV–visible absorption spectra of hALAS2 WT (black), V562A (teal), and M567I (purple). The right panel is zoomed into the region between 300–500 nm. The substituted aldimine and enolimine tautomers absorb around 326 nm and the ketoenamine tautomer absorbs near 424 nm. (B) Fluorescence emission spectra for WT, V562A, and M567I hALAS2 proteins, colored as in panel (A), with excitation at 326 nm. The single peak at 380 nm is indicative of the inactive substituted aldimine species. (C) The ratio of the active ketoenamine population compared to the total PLP bound was determined by the area under the curve of the absorption spectra (**p = 0.0075, ****p < 0.0001).

Due to the differential change in protein stability as a response to exogenous cofactor, we sought to measure the nature of the bound cofactor in the disease variants. Previous studies showed that the hALAS2 C-terminus can affect PLP binding.^52^ To activate the enzyme for catalysis, the PLP cofactor forms a covalent Schiff base with a conserved lysine residue in the active site (K391 in hALAS2).^53^ This internal aldimine linkage can exist as a mixture of tautomers consisting of the substituted aldimine, enolimine, or ketoenamine forms.^54^ Notably, the substituted aldimine species represents a catalytically inactive orientation. The enolimine and ketoenamine forms are in the catalytically active orientation with respect to their hybridization state. The identity of the bound cofactor can be determined with UV–visible and fluorescence spectroscopy. All proteins were purified as holoenzymes bound to steady-state levels of PLP and assayed for the respective tautomer spectral signatures. The WT and variant enzymes displayed two peaks characteristic of a mixture of tautomers (Figure 3A). The first peak centered around 326 nm corresponded to either the inactive substituted aldimine or the active enolimine species, and the second peak at 424 nm represented the active ketoenamine tautomer. For comparison, we purified the hALAS2 G398D variant, which is unable to bind PLP due to steric occlusion of the active site, and showed no absorbance at these characteristic wavelengths (Figure S3). Using fluorescence spectroscopy with excitation at 326 nm, the presence of a single peak at 380 nm represents the substituted aldimine, whereas the enolimine species would emit near 520 nm. Similar to previous reports, we confirmed the peak at 326 nm solely contained the inactive substituted aldimine tautomer (Figure 3B). Although both XLSA variants exhibited similar UV–visible absorbance and fluorescence profiles compared to WT, the relative amounts of the inactive versus active tautomers varied (Figure 3C). Approximately 59% of the total PLP bound to WT hALAS2 was in the active ketoenamine conformation. In contrast, the V562A variant contained a significantly higher proportion of ketoenamine (68%), while the M567I variant only contained 54% of its bound cofactor in the active conformation. Together, these data suggest that steady-state PLP binding is impacted in both C-terminal mutations but in opposite ways.

### XLSA Variant Activity Similarly Responds to PLP

**Figure 4:**
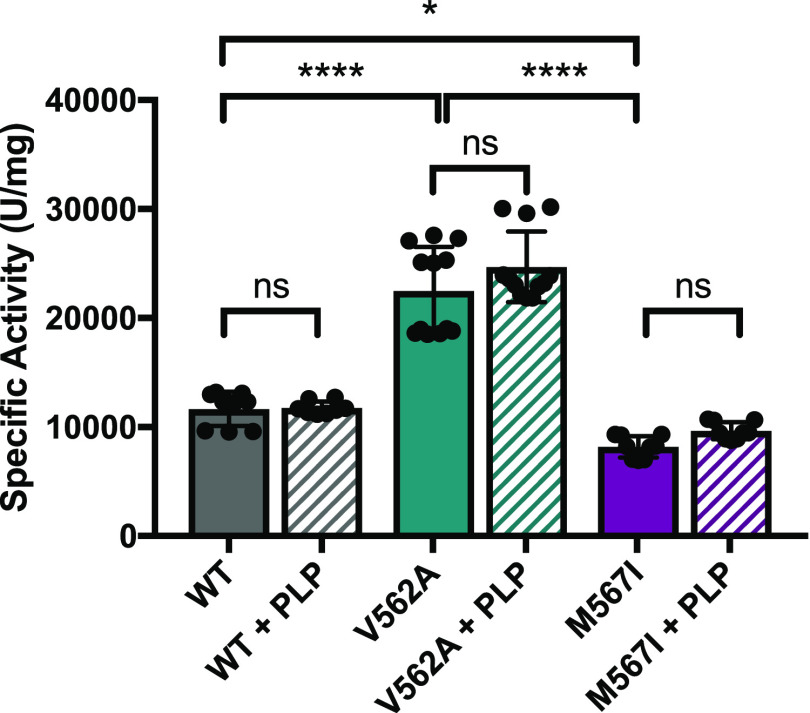
Steady-state activity of hALAS2 variants in the absence and presence of exogenous PLP. Maximal enzyme activity for hALAS2 variants was determined under saturating substrate concentrations in the absence (solid bars) or presence (hatched bars) of exogenous PLP. The addition of excess PLP did not affect hALAS2 WT (gray), V562A (teal), or M567I (purple) activity (*p = 0.0477, ****p < 0.0001, ns: not significant).

Due to changes in enzyme stability and cofactor binding orientation, we also determined if the variant proteins displayed a different response to PLP supplementation. Steady-state enzyme activity was measured before and after the addition of saturating PLP (Figures 4, S4, Table 1). None of the hALAS2 variants displayed a significant increase in activity in the presence of 100-fold excess PLP. However, the V562A variant had significantly higher activity compared to WT, whereas the M567I variant had slightly decreased activity. These results are consistent with a previous report that identified similar trends irrespective of the presence of PLP.^43^

**Table 1:** Specific Activity of hALAS2 Variants with Saturating PLP

	no PLP	percent change^a^	plus PLP (10 μM)	percent change^a^
WT	11700 ± 1590		11,800 ± 596	
V562A	22500 ± 4010	+92%	24,700 ± 3250	+109%
M567I	8170 ± 991	–30%	9660 ± 795	–18%

a Compared to WT.

**Figure 5:**
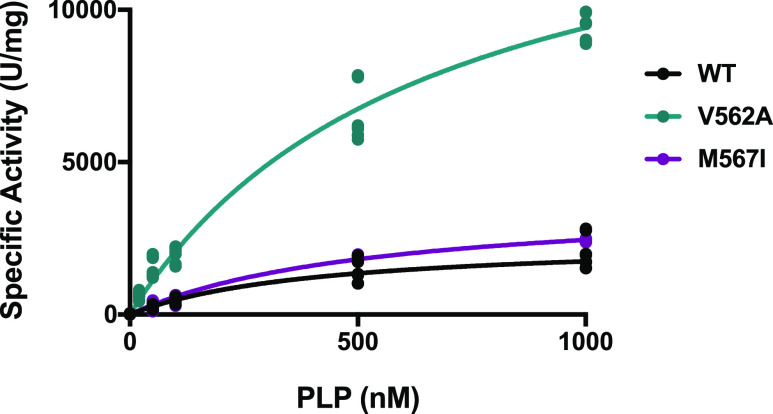
PLP binding kinetics for hALAS2 variants. The activity of the hALAS2 apoenzyme preparations was determined as a function of increasing PLP concentration. Data were normalized by subtracting the signal in the presence of 0 nM PLP. Each experiment was performed with a minimum of three biological replicates, each containing three technical replicates (data points represent the technical replicates).

It is possible that the differences in cofactor binding mode and enzyme responsiveness could be reflected in changes in the apparent PLP binding affinity. To this end, we measured ALA production as a function of increasing amounts of PLP added to the apoenzymes (Figure 5). WT hALAS2 binds PLP with a high nanomolar binding constant of approximately 400 nM. Although both variants also displayed nanomolar PLP binding affinity, the values were elevated compared to WT. Despite the higher *K*_M_, the M567I variant had a faster catalytic rate (*k*_cat_), which resulted in a comparable catalytic efficiency compared to WT. Interestingly, the V562A variant had the largest defect in affinity but ∼6 times higher maximum enzyme velocity and faster *k*_cat_ compared to WT. This variant was also an order of magnitude more efficient than WT (Table 2). Together, these data indicate that the changes in the mode of PLP binding are not due to gross defects in initial PLP binding or activity in the presence of saturating substrate.

**Table 2:** Kinetic Parameters of hALAS2 Variants

	WT	V562A	M567I^a^
PLP			
*V*_max_ (U/mg)^b^	2470 ± 160	15600 ± 850	3780 ± 31
*K*_M_ (nM)	413 ± 63	653 ± 74	542 ± 17
*k*_cat_ (sec^–1^)	3.4 × 10^–4^ ± 2 × 10^–5^	2.1 × 10^–3^ ± 1 × 10^–4^	5.17 × 10^–4^ ± 4 × 10^–6^
*k*_cat_/*K*_M_ (sec^–1^ M^–1^)	823	3220	1060
Glycine			
*V*_max_ (U/mg)	12800 ± 333	28500 ± 824	13700 ± 2550
*K*_M_ (mM)	10.1 ± 0.8	23 ± 2	26 ± 17
*k*_cat_ (sec^–1^)	1.75 × 10^–3^ ± 5 × 10^–5^	3.9 × 10^–3^ ± 1 × 10^–4^	1.45 × 10^–3^ ± 5 × 10^–5^
*k*_cat_/*K*_M_ (sec^–1^ M^–1^)	0.173	0.169	0.0558
Succinyl-CoA			
*V*_max_ (U/mg)	14900 ± 384	26400 ± 1090	9040 ± 329
*K*_M_ (μM)	108 ± 8	272 ± 26	41 ± 5
*k*_cat_ (sec^–1^)	2.04 × 10^–3^ ± 5 × 10^–6^	3.6 × 10^–3^ ± 1 × 10^–4^	1.16 × 10^–3^ ± 2 × 10^–5^
*k*_cat_/*K*_M_ (sec^–1^ M^–1^)	18.9	13.2	28.3

a Allosteric sigmoidal fit: Glycine n_h_ = 0.7 ± 0.1; succinyl-CoA n_h_ = 0.78 ± 0.07

b U measured as nmol of ALA produced per hour

### Diminished Substrate Binding Reveals New Clues into the Molecular Basis for hALAS2 Dysfunction

**Figure 6:**
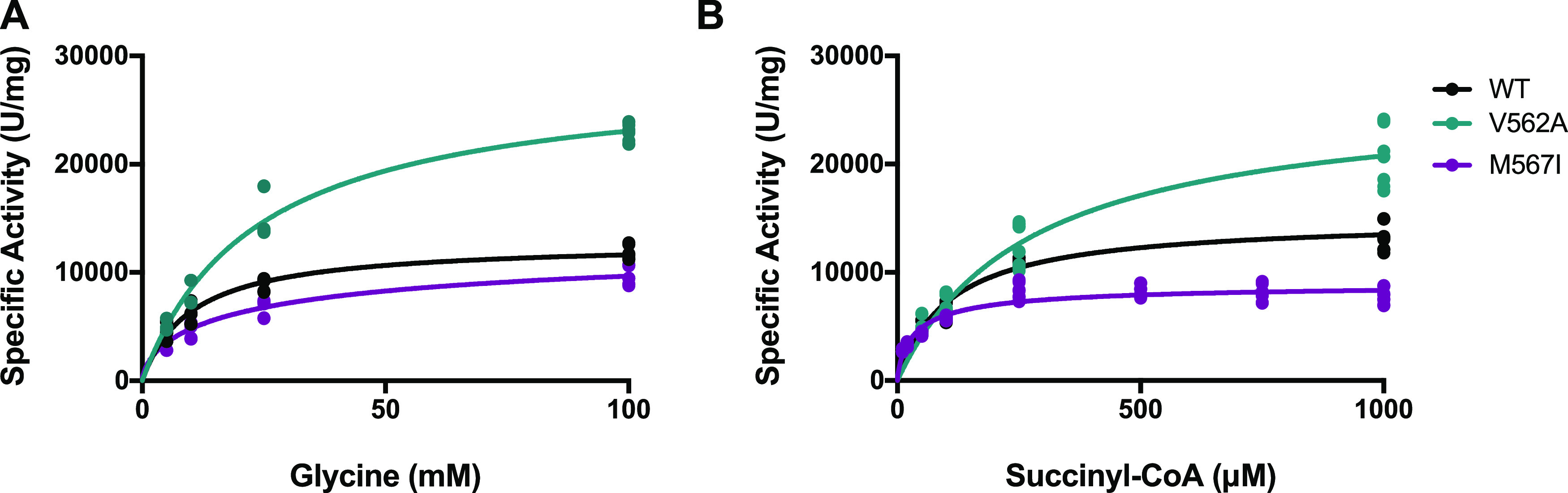
Substrate binding kinetics for hALAS2 variants. (A) The activity of the hALAS2 variants was determined as a function of varied glycine (A) or succinyl-CoA (B) concentrations. Data were fit with a Michaelis–Menten model for hALAS2 WT and V562A and with an allosteric sigmoidal model for hALAS2 M567I. Each experiment was performed with a minimum of three biological replicates, each containing three technical replicates (data points represent the technical replicates).

It is known that some XLSA-associated hALAS2 mutations located both in the catalytic core and the C-terminal extension have diminished binding and response to substrates.^40^ Thus, PLP binding may not be the sole determinant of enzyme function. We measured enzyme activity as a function of increasing concentrations of either glycine or succinyl-CoA in the presence of saturating PLP (Figure 6, Table 2). Concerning glycine binding, hALAS2 V562A had approximately twofold higher *K*_M_ and *V*_max_ compared to WT. Despite the higher *K*_M_, the faster *k*_cat_ resulted in a catalytic efficiency similar to WT. For succinyl-CoA, the *K*_M_ was 2.5 times higher than WT, which resulted in impaired catalytic efficiency despite V562A having a faster catalytic rate. Therefore, it appears that the higher activity (*V*_max_) was at the expense of catalytic efficiency due to a greatly diminished binding affinity for succinyl-CoA. One potential explanation for this behavior is the reaction transition state may be significantly favored over product formation at lower substrate concentrations.

Notably, the M567I variant displayed contrasting enzymatic characteristics in comparison to both WT and V562A. Although the M567I glycine *K*_M_ was elevated like V562A, the *V*_max_ was significantly closer to WT and the *k*_cat_ was diminished. In combination, this led to very inefficient catalysis concerning glycine. The M567I variant bound succinyl-CoA more readily, with approximately 2.6-fold lower *K*_M_ versus WT, although the catalytic efficiency remained unaffected. Importantly, M567I exhibited negative cooperativity in binding both glycine and succinyl-CoA with Hill coefficients of 0.7 ± 0.1 and 0.78 ± 0.07, respectively. This would indicate that the binding of substrates at one of the two dimer active sites would disfavor binding at the second site, leading to slower reaction velocity and turnover for M567I.

### C-terminal Variants Highlight Distinct Mechanisms of Enzyme Dysfunction

**Figure 7:**
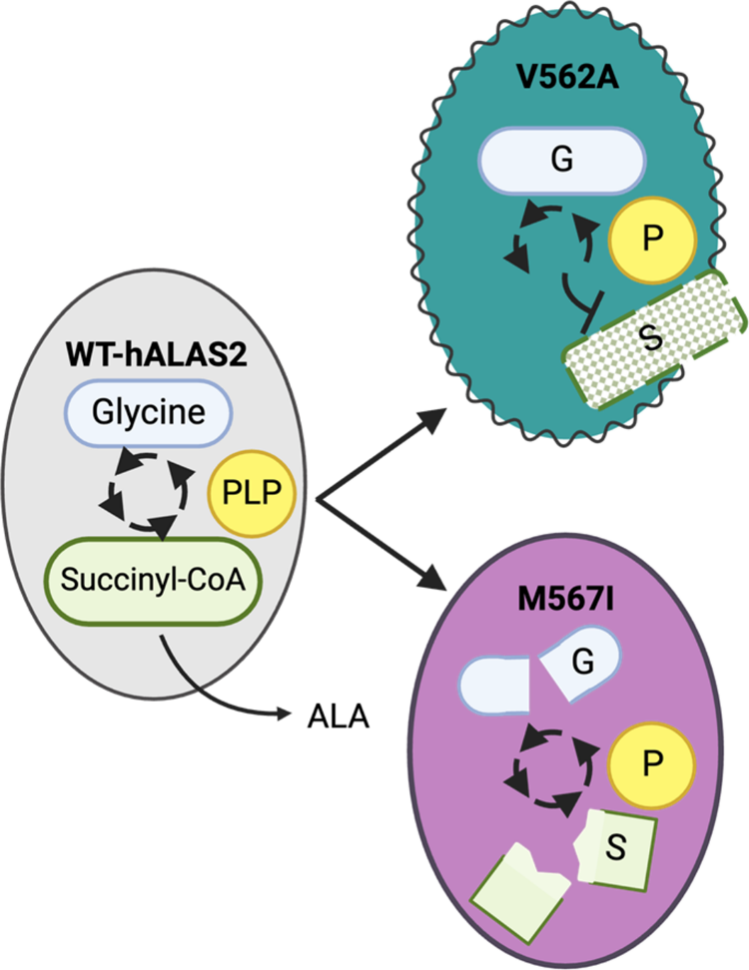
hALAS2 mutations alter enzyme activity via different mechanisms. Summary of the molecular impact of hALAS2 C-terminal mutations on enzyme structure and function. ALAS2 enzymes are depicted as either a large gray (WT), teal (V562A), or purple (M567I) oval. The substrates are represented in light blue (glycine) and green (succinyl-CoA) shapes and the PLP cofactor is represented as a yellow circle. The V562A variant displays decreased stability (blurred outline), abrogated succinyl-CoA binding affinity (hatched substrate rectangle), and catalytic efficiency. In contrast, the M567I variant binds both substrates with negative cooperativity (broken shapes) and has impaired enzyme activity, which may be the primary driver of hALAS2 loss of function underlying X-linked sideroblastic anemia.

Our results indicate that both the V562A and M567I variants have divergent mechanisms of hALAS2 dysfunction resulting from different alterations in cofactor and substrate binding in each variant with the added element of destabilization for V562A (Figure 7). The hALAS2 M567I variant has decreased catalytic activity compared to WT, which appears to primarily be due to a change in intersubunit communication that disfavors the simultaneous engagement of both active sites. One possibility is that the introduction of a smaller residue at this position may introduce new hydrophobic interactions with the enzyme core that could block the active site. Future investigation may reveal the presence of unidentified allosteric hotspots or interaction interfaces that produce a functional conformational change not yet predicted. Curiously, the hALAS2 V562A variant shows enhanced activity in several ways that are contrary to its role in XLSA. First, this enzyme binds PLP in a mode that is competent for catalysis to a higher extent than WT. This was observed by the higher proportion of PLP in the active conformation. Additionally, this variant has significantly higher enzyme velocity in the presence of saturating amounts of cofactor or substrate. Despite these apparent benefits, the V562A mutation results in a decreased cellular protein half-life.^43^ Consistent with the cellular data, our results with thermal stability show that V562A is destabilized compared to WT. Likewise, we show that this variant is a catalytically inefficient enzyme and the succinyl-CoA substrate binds with a weaker affinity compared to WT. The combinatorial mechanism between impaired succinyl-CoA binding coupled with a destabilized enzyme for V562A reveals the molecular defects exhibited by this XLSA variant.

The *in vitro* defects reported here also reveal how individual ALAS2 mutations may have diverse mechanisms leading to the pathophysiology of XLSA. First, alterations in substrate binding affinity for variant enzymes reported here may only initiate pathogenic consequences in instances of decreased substrate availability or metabolic stress. For example, lower glycine levels were found to be correlated with type 2 diabetes, which may have a significant effect in the M567I background.^55^ The increased succinyl-CoA *K*_M_ for the V562A variant may have a more pronounced impact on erythropoiesis *in vivo*, where the concentration of succinyl-CoA can vary significantly. Decreased succinyl-CoA levels were identified in patients with chronic heart failure, which could affect disease presentation and severity.^56^ Additionally, changes in enzyme turnover may have a different physiological impact depending on the stage of erythroid maturation that is affected. A recent clinical report of two patients with ALAS2 mutations resulting in XLSA identified changes in serum hepcidin and erythroferrone levels, highlighting the intimate relationship between iron homeostasis and erythropoiesis, which may also be skewed by ALAS2 dysfunction.^22^ Historically, a significant challenge in determining specific XLSA disease etiology was a lack of model systems that adequately recapitulate the phenotypes found in humans. More recently, advances in both cell and murine-based models that address this previous limitation provide an avenue for future investigation of ALAS2 C-terminal variants.^57−59^

One outstanding question in the field involves determining how C-terminal perturbations can lead to either loss or gain of function. Based on the WT hALAS2 crystal structure, the C-terminal domain is positioned near the active site in a manner that would sterically hinder cofactor and substrate binding.^45^ This autoinhibitory conformation is stabilized by electrostatic interactions between Arg511 in the catalytic core and Glu569 in the C-terminal extension (Figure S5). Ablation of this interaction leads to an increase in catalytic activity.^45^ Although residues Val562 and Met567 are located immediately upstream of Glu569, it is unclear how the mutations investigated in this study could perturb the C-terminus to yield the opposite phenotype because they do not directly impact the electrostatic interaction between Arg511 and Glu569. The computationally predicted models support a propensity for the C-terminus to be highly flexible and the crystal structure may represent one of the low-energy conformations (Figure 1). Additional structural changes could also be conferred by different protein or small molecule interactions, but these would be difficult to predict computationally. For example, several metabolic networks are reported to be modulated by multiprotein complexes, including mitochondrial respiration,^60^ the TCA cycle,^61−65^ glycolysis,^66−69^ urea cycle,^70,71^ fatty acid metabolism,^72^ and purine synthesis.^73−77^ Heme biosynthesis is also putatively controlled by the assembly of multiple mitochondrial proteins, including ALAS2.^37,38^ A previous report identified certain ALAS2 C-terminal mutations associated with XLSA (ALAS2 M567V and S568G) that also abrogate binding to the TCA cycle enzyme SCS *in vitro*.^40^ Additionally, heme itself may modulate ALAS2 function and interactions. It is established that heme binding impedes ALAS translocation into the mitochondrial matrix,^78^ as well as altering protein interactions and degradation for human ALAS1.^79−81^ Future *in vivo* work will be necessary to parse apart the impact of ALAS2 C-terminal mutations in biomolecular protein assembly and whether changes in protein interactions mediated by these diverse loss-of-function mutations are either correlative or causative for disease.

## Conclusions

ALAS is often referred to as the gatekeeper of heme biosynthesis because it controls the first committed step of heme production.^82^ In addition, the expression of certain heme biosynthetic genes is dependent on proper heme production, which is largely informed by the expression of ALAS2. This further highlights its critical role in heme production.^83^ Thus, perturbations in hALAS2 structure and function can have diverse impacts on heme synthesis and erythropoiesis. Specifically, the hALAS2 C-terminal extension represents a key means of enzyme regulation as multiple disease variants with opposing phenotypes are located in this domain. Here, we investigated two seemingly mild hALAS2 C-terminal mutations that maintain the hydrophobic character of the WT amino acid but slightly perturb side chain size and/or charge. These mutations did not cause gross structural defects, but they impacted protein stability and substrate binding in distinct ways.

Although our work gives new insights into the molecular basis for disease due to hALAS2 C-terminal perturbations, we also acknowledge that multiple molecular and cellular mechanisms work in concert to yield disease. Importantly, these mechanisms are not mutually exclusive or limited to C-terminal variants. For example, the hALAS2 R452C variant was shown to have impaired PLP and succinyl-CoA affinity.^40^ Other variants like hALAS2 S568G have altered substrate binding but may also have an altered protein–protein interaction profile.^40^ Thus, depending on the context and considering other patient-specific genetic and epigenetic factors, an individual mutation may have pleiotropic effects. Our work contributes to the understanding of how the eukaryote-specific C-terminal domain affects ALAS2 function to regulate heme biosynthesis and erythropoiesis. Thus, future work may reveal how to adapt the C-terminal extension structure or biomolecular interactions to ensure optimal ALAS2 function.

## Supporting Information Available

AlphaFold models and corresponding pLDDT scores; Western blot of purified ALAS2 variants; UV–visible absorbance spectrum of hALAS2 G398D; multiple comparison statistical analysis of *in vitro* ALAS2 activity; depiction of the inhibitory hALAS2 C-terminal extension conformation observed in the crystal structure; primer sequences used for site-directed mutagenesis (PDF)

## Abbreviations

ALA: aminolevulinic acid
ALAS: aminolevulinic acid synthase
DTT: dithiothreitol
pLDDT: predicted local distance difference test
PLP: pyridoxal 5′-phosphate
SCS: succinyl-CoA synthetase
Succinyl-CoA: succinyl-coenzyme A
TCEP: tris(2-carboxyethyl)phosphine
XLP: X-linked protoporphyria
XLSA: X-linked sideroblastic anemia
