# Supplementary material for: Elucidating the Role of Human ALAS2 C-terminal Mutations Resulting in Loss of Function and Disease
Source: Biochemistry. 2024 Jun 18;63(13):1636–46. doi: 10.1021/acs.biochem.4c00066 (PMC11223264; doi:10.1021/acs.biochem.4c00066)
Supplement: Supplementary file 1 — bi4c00066_si_001.pdf [file bi4c00066_si_001.pdf]

Supplementary Information

Elucidating the role of human ALAS2 C-terminal mutations resulting in loss-of-function and disease

Jessica L. Taylor,[a] Pedro H. Ayres-Galhardo,[a] and Breann L. Brown[a,b]*

[a]Department of Biochemistry, [b]Center for Structural Biology, Vanderbilt University School of Medicine, Nashville, TN 37232

*For correspondence:
Breann L. Brown, Ph.D.
2215 Garland Ave
Nashville, TN 37232
615-343-1632
breann.brown@vanderbilt.edu

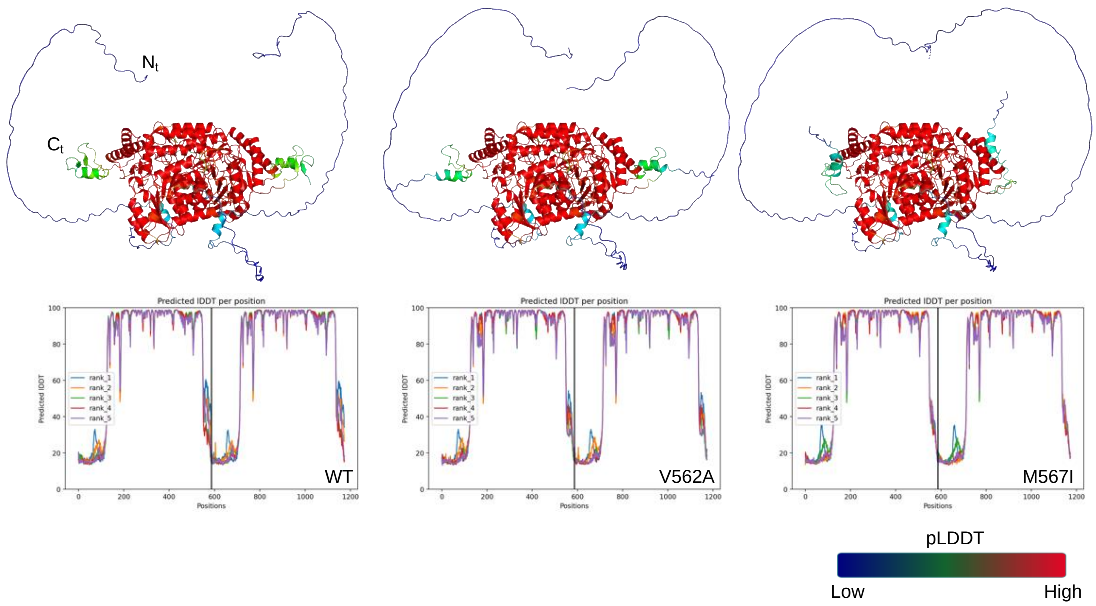

Figure S1. AlphaFold Multimer confidence analysis. AlphaFold models colored by pLDDT scores (top panel) with corresponding per residue plots shown below. A higher pLDDT score (red) corresponds to a higher confidence in prediction.

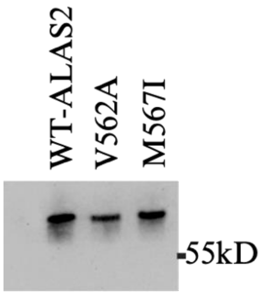

Figure S2. Western blot of WT ALAS2 and variants indicate that the purified proteins have not undergone C-terminal proteolytic cleavage. The anti-hALAS2 antibody (Abcam ab184964) recognizes an epitope within the terminal 14 amino acids (residues 574-587). The immunoblot results were confirmed with LC-MS/MS. Approximately 77ng of each sample was loaded onto a 7.5% TGX Polyacrylamide Protean Gel (BioRad). After electrophoresis, the samples were transferred to a PVDF membrane followed by blocking at room temperature for 2 hrs in buffer (PBS with 0.05% Tween-20 and 3% milk). The membrane was incubated in a 1:3000 dilution of primary antibody at RT for 1hr, followed by a 1 hr RT incubation in Goat anti-Rabbit IgG secondary antibody (Invitrogen) at 1:15,000 dilution. The membrane was developed using CDP Chemiluminescent Substrate (Sigma).

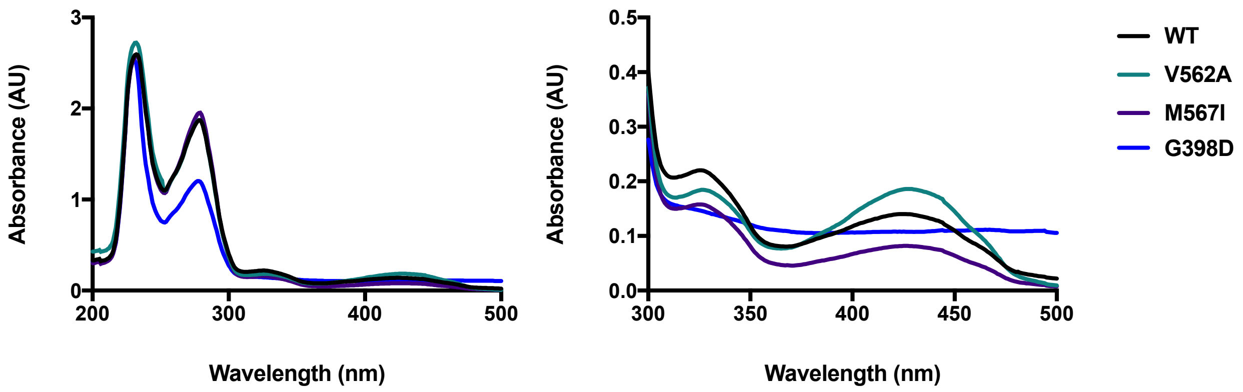

Figure S3. UV-Visible absorption spectra of hALAS2 WT (black), V562A (teal), M567I (purple), and G398D (blue). The right panel is zoomed into the region between 300-500 nm. The hALAS2 G398D variant does not bind PLP due to steric occlusion of the PLP binding pocket.

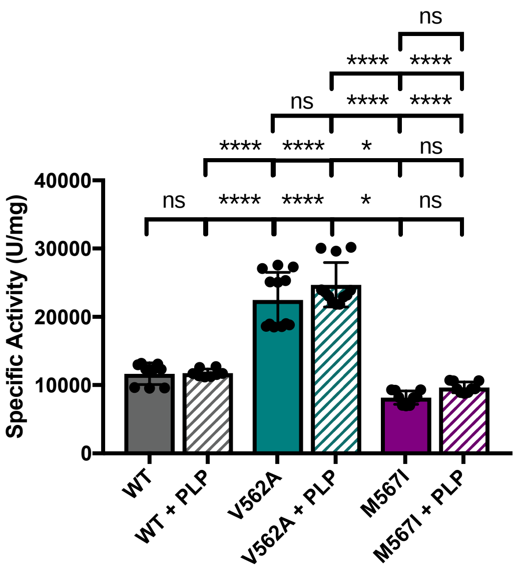

Figure S4. Complete statistical analysis of the enzyme activity for hALAS2 variants determined under saturating substrate concentrations in the absence (solid bars) or presence (hatched bars) of exogenous PLP. (*p<0.05, ****p<0.0001, ns: not significant).

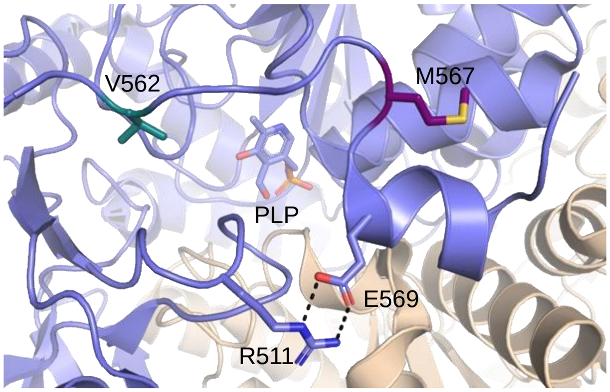

Figure S5. The hALAS2 C-terminal extension is stabilized in an inhibitory conformation via the interaction between residues R511 and E569. Residues V562 and M567 are colored as in the text. All residues and the PLP cofactor are depicted as stick representations.

Table S1. Primer sequences used for site-directed mutagenesis

**Variant**	**Primer Sequence (5'- 3')**
G398D	Forward TTTGGCTGTGTGGGCGATTACATTGCCAGCACC<br>Reverse GGTGCTGGCAATGTAATCGCCCACACAGCCAAA
V562A	Forward TTCTGTCGCCGTCCTGCGCACTTTGAGCTCATG<br>Reverse CATGAGCTCAAAGTGCGCAGGACGGCGACAGAA
M567I	Forward CCTGTACACTTTGAGCTCATTAGTGAGTGG<br>Reverse GTAGGAACGTTCCCACTCACTAATGAGCTC
